# (H)IF applicable: promotion of neurogenesis by induced HIF-2 signalling after ischaemia

**DOI:** 10.1007/s00424-021-02600-8

**Published:** 2021-07-12

**Authors:** Tristan Leu, Joachim Fandrey, Timm Schreiber

**Affiliations:** 1 Institute of Physiology, University Duisburg-Essen, 45147 Essen, Germany; 2 Institute of Physiology, Pathophysiology and Toxicology and Center for Biomedical Education and Research (ZBAF), University of Witten/Herdecke, 58453 Witten, Germany

**Keywords:** HIF-2, Ischaemia, Brain, Neurogenesis, Regeneration

## Abstract

HIF-2 represents a tissue-specific isoform of the hypoxia-inducible factors (HIFs) which regulate oxygen homeostasis in the cell. In acute oxygen deficiency, HIF transcription factors ensure the timely restoration of adequate oxygen supply. Particularly in medical conditions such as stroke, which have a high mortality risk due to ischaemic brain damage, rapid recovery of oxygen supply is of extraordinary importance. Nevertheless, the endogenous mechanisms are often not sufficient to respond to severe hypoxic stress with restoring oxygenation and fail to protect the tissue. Herein, we analysed murine neurospheres without functioning HIF-2α and found that special importance in the differentiation of neurons can be attributed to HIF-2 in the brain. Other processes, such as cell migration and signal transduction of different signalling pathways, appear to be mediated to some extent via HIF-2 and illustrate the function of HIF-2 in brain remodelling. Without hypoxic stress, HIF-2 in the brain presumably focuses on the fine-tuning of the neural network. However, a therapeutically increase of HIF-2 has the potential to regenerate or replace destroyed brain tissue and help minimize the consequences of an ischaemic stroke.

## Introduction

Neurological diseases, especially ischaemic stroke, are often associated with the loss of neurons. However, neurogenesis is limited to few areas in the brain, such as the subventricular zone (SVZ), and stem cells need to migrate to the damaged area to replace lost cells. Moreover, only a small proportion of the newly generated neurons seem to survive permanently, which is probably due to unfavourable environmental factors, insufficient connections to supporting cells and excessive confrontation with harmful processes emanating from the damaged tissue [2]. Thus, although certain regeneration mechanisms exist in our brain and the proliferation, neurogenesis and migration of neural precursor cells in certain neurogenic regions increase after damage [31], a longer survival time and integration into the existing system of the damaged region are needed. Interestingly, many of the endogenous processes that influence the repair of cerebral damage are related to the hypoxia-inducible factor (HIF) [6, 8, 14].

HIF is a heterodimer, whose complexity comprises a constitutively expressed HIF-β subunit and an O_2_-regulated HIF-α subunit [42]. So far, three different α subunits were identified. These include HIF-1α, HIF-2α and HIF-3α, whereby just for HIF-1α and HIF-2α, it is known that they act as transcription factors by dimerizing with HIF-ß [5]. In the presence of O_2_ in well-oxygenated tissue_,_ continuously synthesized HIF-αs are hydroxylated by prolyl-4-hydroxylase domain proteins (PHDs) [12] and ultimately degraded by the proteasome. Since PHDs require oxygen for their activity, hypoxia reduces hydroxylation of HIF-αs and allows hypoxic accumulation to form more HIF complexes with the constitutive HIF-ß.

The activation of the HIF pathway under physiological and hypoxic conditions plays an essential role in the development of the central nervous system. With regard to neural cell development in zebrafish, HIF-2 has a protective effect on neuronal stem cells while also promoting neurogenesis [21]. HIF-2 also influences the production of apoptosis inhibitors and controls angiogenesis throughout the brain. This mainly affects the physiological formation of new nerve cells which is a relevant factor in recovering from pathologies such as an ischaemic stroke [28].

Therefore, HIF is particularly relevant in neurodegenerative brain diseases. In a mouse model of ischaemic stroke, a combined neuronal knockout of HIF-1α and HIF-2α resulted in reduced infarct size in the early acute phase during the first 24 h of reperfusion; this effect was lost after 72 h of reperfusion when no differences in infarct size were seen [22]. A reduced expression of anti-survival genes by HIF in the early acute phase might have led to increased apoptotic cell death and reduced angiogenesis. Interestingly, the enhanced accumulation of HIF by a knockout of PHD2 in neurons reduced the infarct size in a mouse stroke model by more than 50% [22]. This implies that special emphasis should be placed on the timing and cell type–specific activation or inhibition of HIF-regulated cytoprotective factors to consider HIF-controlled treatment options in stroke therapy. 

Due to the neuroprotective effects of HIF-2 described above, we focused on the specific role of HIF-2 during the regeneration from ischaemic brain damage to reveal its role in responding to local or systemic oxygen deficiency in the brain.

## Experimental procedures

### Animals

All experiments were performed with male and female C57BL/6 J mice. To obtain mice with a neuronal-specific *Hif-2α*, knockout mice homozygous for loxP sites covering exon 2 of the *Hif-2α* gene which encodes for the DNA binding domain of the transcription factor (*Hif-2α*^+*fl/*+*fl*^, purchased from the Jackson Laboratory, Bar Harbor, ME, USA) were crossed with mice heterozygously expressing Cre recombinase under the control of the nestin promoter (B6.Cg-Tg(Nes-cre)1Kln/J, purchased from the Jackson Laboratory, Bar Harbor, ME, USA). Nestin is already expressed in neuroepithelial cells that form the neural plate and then the neural tube [16]. As a result, all cell types in the brain carry the knockout, since all cell types descend from these cells. Littermates negative for CRE recombinase but *Hif-2α*^+*fl/*+*fl*^ served as control animals. All animals showed a physiological habitus and normal breeding behaviour. Complete pelleted feed and drinking water were administered ad libitum. The keeping and breeding of the animals took place in compliance with the German law for animal welfare and was approved by the State Agency for Nature, Environment and Consumer Protection North Rhine-Westphalia (file reference, 84–02. 04. 2016. A173).

### Cell culture

To obtain neural stem and progenitor cells, mice aged p1–p3 were killed by decapitation, and the head was transferred to sterile PBS. Further preparations were carried out in minimal essential medium (MEM; Thermo Fisher, Waltham, MA, USA). Cranial skin, the skullcap and the meninges were carefully removed, and the cerebral cortices were isolated. Both hemispheres were enzymatically digested with 30 U/mL papain (Worthington, Freehold, NJ, USA) for 20 min at 37° C to obtain single-cell suspensions. Subsequently, 1 ml of the supernatant was discarded, and digestion was terminated by adding 1 ml of ovomucoid (1 mg/ml trypsin inhibitor (Merck KGaA, Darmstadt, Germany), 50 μg/ml BSA and 40 μg/ml DNaseI (Worthington, Freehold, NJ, USA) in MEM). Cells were centrifuged for 5 min at 300 g, the supernatant was completely discarded, and the resulting cell pellet dissolved in 1 ml neurosphere medium (DMEM/F-12 (1:1, Thermo Fisher, Waltham, MA, USA) containing 0.2 mg/ml L-glutamine (Merck KGaA, Darmstadt, Germany), 2% v/v B27 supplement (Thermo Fisher, Waltham, MA, USA), 100 U/ml penicillin and 100 μg/ml streptomycin (both Merck KGaA, Darmstadt, Germany)). Cells were cultivated as free-floating neurospheres in T25 flasks (bulk culture) at a density of 10^5^ cells/ml in neurosphere medium in the presence of 20 ng/mL epidermal growth factor (EGF) and basic fibroblast growth factor (bFGF, PreproTech GmbH, Hamburg, Germany) at 37° C in 20.9% O_2_/5% CO_2._ The neurospheres were cultivated for 12–16 days, changing half of the medium every 3–4 days supplemented with 20 ng/mL EGF and bFGF.

### Oxygen–glucose deprivation

To simulate an ischaemic stroke, neurospheres were placed in glucose-free DMEM-F12 medium for 1 to 4 h at 0.2% O_2_ in the hypoxia chamber resulting in oxygen–glucose deprivation (OGD). Neurospheres were lysed for RNA and protein extraction after 60 min each in the corresponding buffer (treatment # 1). For the additional reperfusion, the neurospheres were transferred in neurosphere medium and incubated for 1 h at 20.9% O_2_ (treatment # 2). The migration of the neurospheres was studied with special differentiation medium for 24 h (neurosphere medium containing 1% v/v FCS (Merck KGaA, Darmstadt, Germany); treatment # 3).

### Migration assay

For measurement of the cellular migration properties before and after OGD, the migration and differentiation were initiated in a four-part cell culture dish (Greiner Bio One, Kremsmuenster, Austria) coated with 10 μg/ml poly-ornithine and 10 μg/ml 445 laminin-1 (Merck KGaA, Darmstadt, Germany) in neurosphere medium containing 1% v/v FCS (Merck KGaA, Darmstadt, Germany). Migration distance was measured from the edge of the sphere to the farthest migrated cells at four defined positions per sphere after 24, 48 and 72 h. The initial size of the neurospheres was not included in the migration distance, since the migration speed is independent of the size of the sphere [26].

### Polymerase chain reaction

Total RNA was isolated from neurospheres with the NucleoSpin RNA kit (MACHEREY–NAGEL GmbH & Co. KG, Düren, Germany). cDNA was synthesized using M-MLV reverse Transcriptase (Promega GmbH, Walldorf, Germany), and qPCR analysis was performed with Biozym Blue S’Green qPCR-Kit (Biozym Scientific GmbH, Oldendorf, Germany) on Bio-Rad’s CFX96™ real-time system (Bio-Rad Laboratories GmbH, Feldkirchen, Germany). The following primer pairs were used: *Bcl2* 5′-ATGCCTTTGTGGAACTATATGGC-3′ forward and 5′-GGTATGCACCCAGAGTGATGC-3′ reverse; *Cdk5r1* 5′-CTGTCCCTATCCCCCAGCTAT-3′ forward and 5′-GGCAGCACCGAGATGATGG-3′ reverse; *Cdkn1a* 5′-CGAGAACGGTGGAACTTTGAC-3′ forward and 5′-CCAGGGCTCAGGTAGACCTT-3′ reverse; *Cited2* 5′-CGCCAGGTTTAACAACTCCCA-3′ forward and 5′-TGCTGGTTTGTCCCGTTCAT-3′ reverse; *Cxcl1* 5′-CTGGGATTCACCTCAAGAACATC-3′ forward and 5′-CAGGGTCAAGGCAAGCCTC-3′ reverse; *Epo* 5′-ACTCTCCTTGCTACTGATTCCT-3′ forward and 5′-ATCGTGACATTTTCTGCCTCC-3′ reverse; *Hif-1α* 5′-ACCTTCATCGGAAACTCCAAAG-3′ forward and 5′-CTGTTAGGCTGGGAAAAGTTAGG-3′ reverse; *Hif-2α exon 2* 5′-AGGAGACGGAGGTCTTCTATGA-3′ forward and 5′-ACAGGAGCTTATGTGTCCGA-3′ reverse; *Ldha* 5′-TGTCTCCAGCAAAGACTACTGT-3′ forward and 5′-GACTGTACTTGACAATGTTGGGA-3′ reverse; *NeuroD1* 5′-ATGACCAAATCATACAGCGAGAG-3′ forward and 5′-TCTGCCTCGTGTTCCTCGT-3′ reverse; *Ngf* 5′-CCAGTGAAATTAGGCTCCCTG-3′ forward and 5′-CCTTGGCAAAACCTTTATTGGG-3′ reverse; *Notch1* 5′-GATGGCCTCAATGGGTACAAG-3′ forward and 5′-TCGTTGTTGTTGATGTCACAGT-3′ reverse; *Nrg1* 5′-ATGGAGATTTATCCCCCAGACA-3′ forward and 5′-GTTGAGGCACCCTCTGAGAC-3′ reverse; *Olig2* 5′-TCCCCAGAACCCGATGATCTT-3′ forward and 5′-CGTGGACGAGGACACAGTC-3′ reverse; *Phd2* 5′-TTGTTACCCAGGCAACGGAAC-3′ forward and 5′-CCTTGGCGTCCCAGTCTTT-3′ reverse; *Phd3* 5′-AGGCAATGGTGGCTTGCTATC-3′ forward and 5′-GCGTCCCAATTCTTATTCAGGT-3′ reverse; *Rpl13a* 5′-CTGTGAAGGCATCAACATTTCTG-3′ forward and 5′-GACCACCATCCGCTTTTTCTT-3′ reverse; and *Vegfa* 5′-ACTGGACCCTGGCTTTACTG-3′ forward and 5′-ACTTGATCACTTCATGGGACTTCT-3′ reverse. Genes of interest were normalized to 60S ribosomal protein L13a (Rpl13a) as indicated. Expression was calculated with the 2^−ΔCT^ method.

### Immunofluorescence analysis

For immunofluorescence staining, cells were fixed with 4% PFA. Cells were permeabilized for 10 min in 0.3% PBS-T, non-specific binding sites blocked with 10% goat serum/PBS-T within 60 min, and antibodies were diluted in 0.1% PBS-T. The following antibodies were used: 1:200 anti-β-Tubulin-III IgG (Sigma-Aldrich, Inc., #T2200) and 1:200 Alexa Fluor® 488 goat anti-rabbit IgG (Life Technologies, # A32731) for visualization of neurons; 1:200 anti-glial fibrillary acidic protein (GFAP) (Merck Millipore, #MAB360) and Alexa Fluor® 568 goat anti-mouse IgG (Invitrogen AG, #A11004) for visualization of astrocytes; anti-Marker O4 IgM (R&D Systems, #MAB1326) and Alexa Fluor® 488 goat anti-mouse IgG (Life Technologies, #A11001) for visualization of oligodendrocytes. For additional visualization of the nuclei, cells were covered with DAPI-added Mowiol.

### Western blot

Western blot analysis was performed as previously described [45] with 20 µg protein lysate per line. The following antibodies were used: 1:500 anti-HIF-1α (Cayman Chemical, #10,006,421) and 1:1000 anti-α-Tubulin (Santa Cruz Biotechnology, #sc-8035).

### Statistics

The software GraphPad Prism® version 8.0.2 from GraphPad Software, Inc. was used to evaluate the results. Statistical significance was determined using unpaired, two-tailed Student’s t-test. For all quantified data, mean ± SEM values are presented. A p-value ≤ 0.05 was considered as statistically significant (p ≤ 0.05 = *). Significances in relation to the corresponding control group were marked with a # (p ≤ 0.05 = #).

## Results

**Fig. 1:**
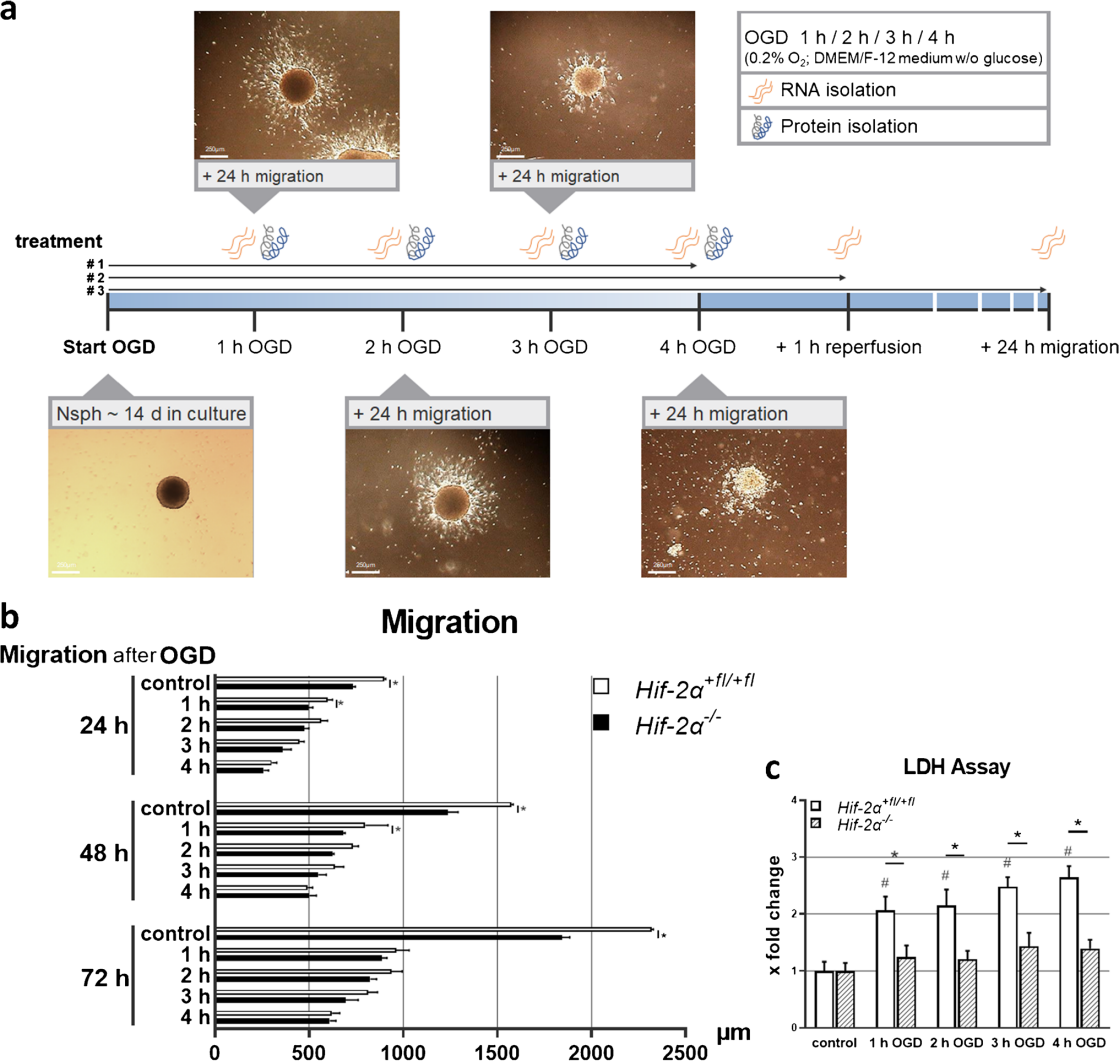
OGD treatment reduces migration and enhances mortality of neural cells. **a** Schematic of OGD treatment simulating an ischemic stroke in vitro with neurospheres. **b** Measurement of migration distances after OGD and 24, 48 and 72 h of migration (n = 3). **c** Measurement of lactate dehydrogenase in the culture medium during OGD for determining mortality rate (n = 5). Data are presented as mean ± SEM; *p < 0.05

To determine the role of HIF-2α in stroke, we used an in vitro neurosphere assay. Neural stem cells (NSCs) were isolated from wild-type mouse pups (*Hif-2α*^+*fl/*+*fl*^) and littermates without active HIF-2α protein (*Hif-2α*^*−/−*^) and grown as neurospheres. Subsequently, spheres were treated with oxygen–glucose deprivation (OGD), as depicted in Fig. 1a, to simulate cerebral ischemia in vitro. OGD time points longer than 4 h were not possible due to complete cell decay under the OGD conditions. To investigate the influence of HIF-2α during reperfusion and regeneration, we reperfused the spheres with normoxia and glucose-containing medium for 1 h, and let the spheres differentiate for up to 72 h.

First, to analyse basic cell properties and to test the function of the cells, we measured the distance that wild-type and *Hif-2α* knockout cells migrated from the spheres after OGD treatment (Fig. 1b). These measurements showed that the cells migrated radially away from the spheres with increasing differentiation time, whereby migration was impaired in *Hif-2α*^*−/−*^ cells. OGD treatment robustly decreased the migration distance up to 75% in wild-type as well as in knockout cells. Interestingly, wild-type cells were more strongly affected than knockout cells, and their advantage in migration steadily disappeared with increasing OGD duration.

Cells are enormously stressed during ischaemia. Therefore, we tested cell viability with an LDH assay after OGD. LDH release in *Hif-2α*^+*fl/*+*fl*^ cultures increased moderately but significantly with ongoing OGD duration (Fig. 1c). Interestingly, the viability of *Hif-2α*^*−/−*^ spheres appeared to be unaffected by OGD.

**Fig. 2:**
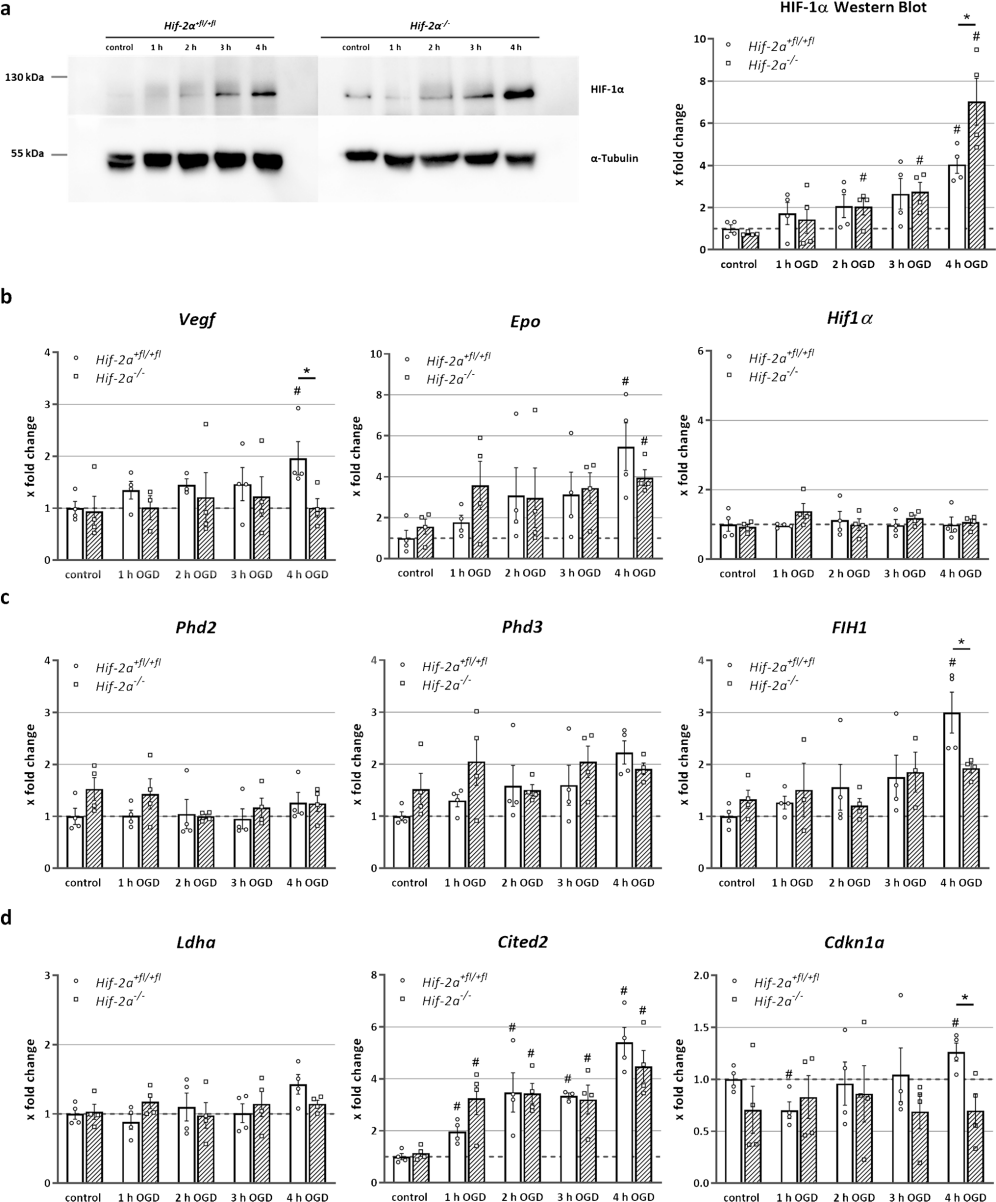
HIF-2 signalling is regulated differently in neural cells. Western blot of HIF-1 and quantitative PCR analysis for specific HIF target genes or HIF signalling regulating genes. Protein and RNA from cells during different OGD time points were used. Genes were quantified and normalized for *Rpl13a* expression. **a** Western Blot analysis of HIF-1α during OGD. **b** Known HIF target genes from tissue other than the brain. **c** Genes that regulate the HIF signalling pathway. **d** Genes potentially regulated by the FIH signalling cascade. Data are presented as mean ± SEM; *p < 0.05; n = 4 with individual data points of each mouse

Various studies have shown that HIF isoforms potentially compensate for each other [36]. Therefore, we analysed HIF-1α protein stabilization after OGD. As expected, HIF-1α was increasingly stabilized during increasing OGD duration. We also found that after 4 h of OGD, the amount of HIF-1α accumulated significantly more in the *Hif-2α*^*−/−*^ cells compared to *Hif-2α*^+*fl/*+*fl*^ cells (Fig. 2 a). *Hif-1α* mRNA expression did not show any differences, neither between *Hif-2α*^+*fl/*+*fl*^ and *Hif-2α*^*−/−*^ nor during OGD.

To verify increased transcriptional activity by HIF-1α accumulation after loss of HIF-2α, we looked for the expression of the known HIF target genes *Vegf* and *Epo*. Here, *Vegf* expression increased steadily during OGD in the wild-type spheres but was unaffected in the knockout cells (Fig. 2b). *Epo* expression rose with ongoing OGD in both genotypes, with stronger increase in the wild-type. HIF-1α seems to compensate for the loss of HIF-2α in the case of ischaemia to some extent, although the known HIF target genes *Epo* and *Vegf* seem to be regulated by HIF-2α not HIF-1α in NSCs. Collectively, the loss of HIF-2α seems to be beneficial for NSCs in the event of ischaemia, presumably due to increased HIF-1α stabilization.

**Fig. 3:**
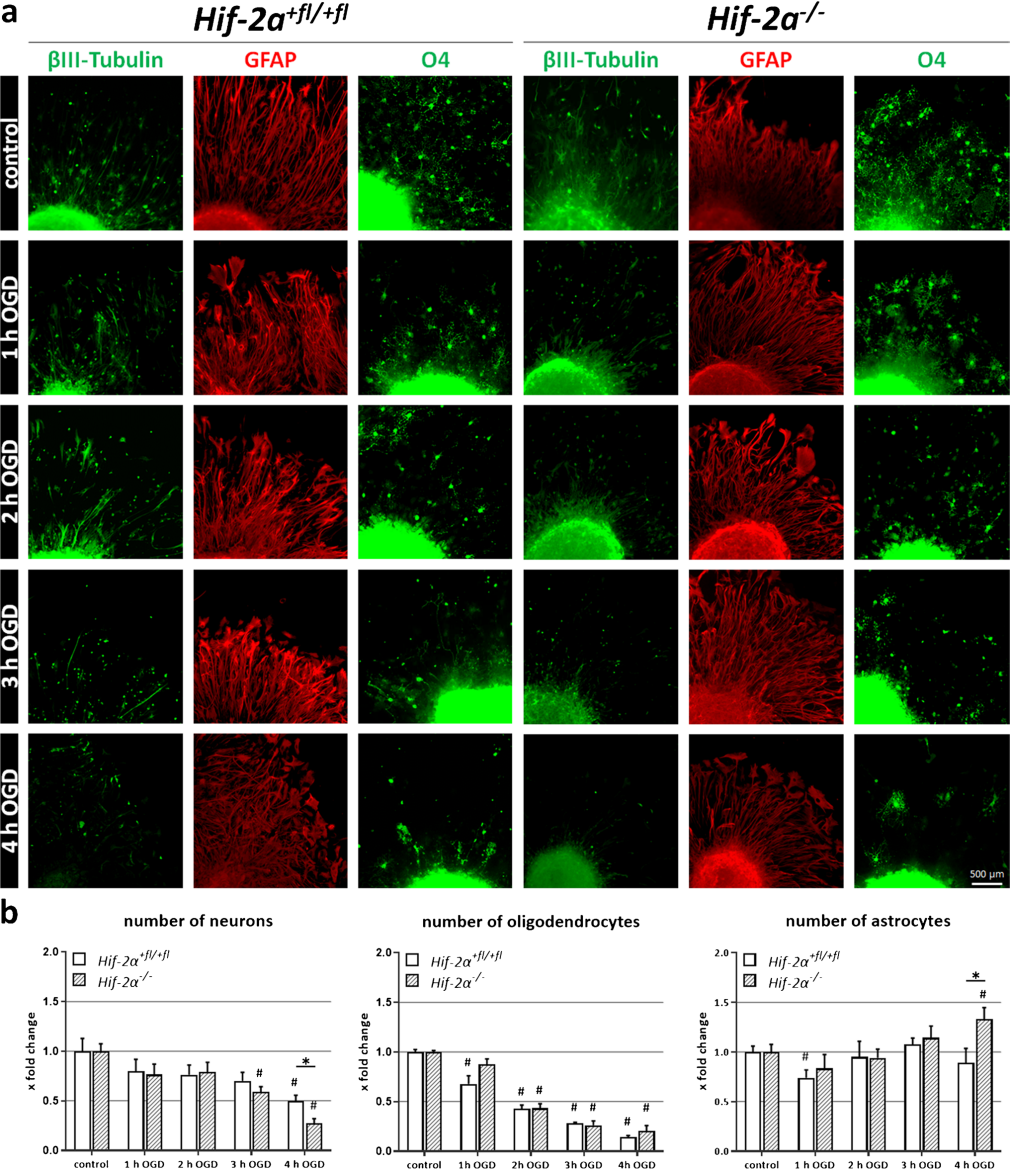
*Hif-2α* knockout in neurospheres leads to significantly less neurons after ischaemia. **a** Immunofluorescence staining of neurons (βIII tubulin), astrocytes (GFAP) and oligodendrocytes (O4) migrated for 24 h from *Hif-2α*^+*fl/*+*fl*^ and *Hif-2α*^*−/−*^ neurospheres. **b** Relative cell number related to control. Data are presented as mean ± SEM; *p < 0.05; n = 5

As loss of HIF-2α changes migration capabilities and OGD susceptibility, we wanted to clarify which cell types are among the migrating cells and how they are affected by OGD. Therefore, we performed immunofluorescence staining after 1-h reperfusion and 72 h of differentiation. The quantification of immunoreactive cells for β(III) tubulin showed that the number of neurons in the migration area decreased in *Hif-2α*^+*fl/*+*fl*^ and *Hif-2α*^*−/−*^ spheres with ongoing OGD duration (Fig. 3). After 4 h of OGD, the number of neurons decreased by roughly 50% in wild-type and 75% in KO spheres. Oligodendrocytes were even more susceptible than neurons; their number decreased by more than 80% in both genotypes after 4 h of OGD, without any difference between KO and WT. The cell count of astrocytes fluctuated around control values until an increase in the *Hif-2α*^*−/−*^ spheres after 4 h of OGD. At this time point, migration behaviour of astrocytes appeared somewhat more disordered, but this was not constantly observed in all neurospheres. Here, we showed that OGD led to a reduced differentiation of NSCs into neurons and oligodendrocytes. The functional loss of HIF-2α caused an even greater reduction of neurons, but to an increased number of astrocytes.

Since a modified *Hif-1α* gene expression was excluded as an explanation for the altered HIF-1α accumulation, the next step was to examine HIF-regulating enzymes, namely, PHD2, PHD3 and FIH1. The constantly expressed *Phd1* was excluded in this study. Expression of *Phd2* and *Phd3* did not differ between *Hif-2α*^+*fl/*+*fl*^ and *Hif-2α*^*−/−*^ and was not regulated during OGD (Fig. 2 c). However, analysis of *FIH1* expression showed a significant increase after 4 h of OGD in *Hif-2α*^+*fl/*+*fl*^ spheres and thus differs significantly from *Hif-2α*^*−/−*^ spheres.

To determine the significance of altered *FIH1* expression, we analysed the potentially FIH-dependent HIF-2 target genes *Ldha, Cited-2* and *Cdkn1a* [9]. *Ldha* expression did not show any changes over the entire OGD period in both genotypes (Fig. 2d). In contrast, *Cited-2* showed a significant increase in *Hif-2α*^+*fl/*+*fl*^ and *Hif-2α*^*−/−*^ spheres already after 1 h of OGD, and this continued to rise during the experiment. *Cdkn1a* expression in the wild-type initially decreased after 1 h of OGD but increased afterwards. In the *Hif-2α*^*−/−*^ spheres, on the other hand, the expression did *not* differ throughout the entire duration of the experiment. Thus, of the three investigated genes, only *Cdkn1a* seems to be regulated by HIF-2.

**Fig. 4:**
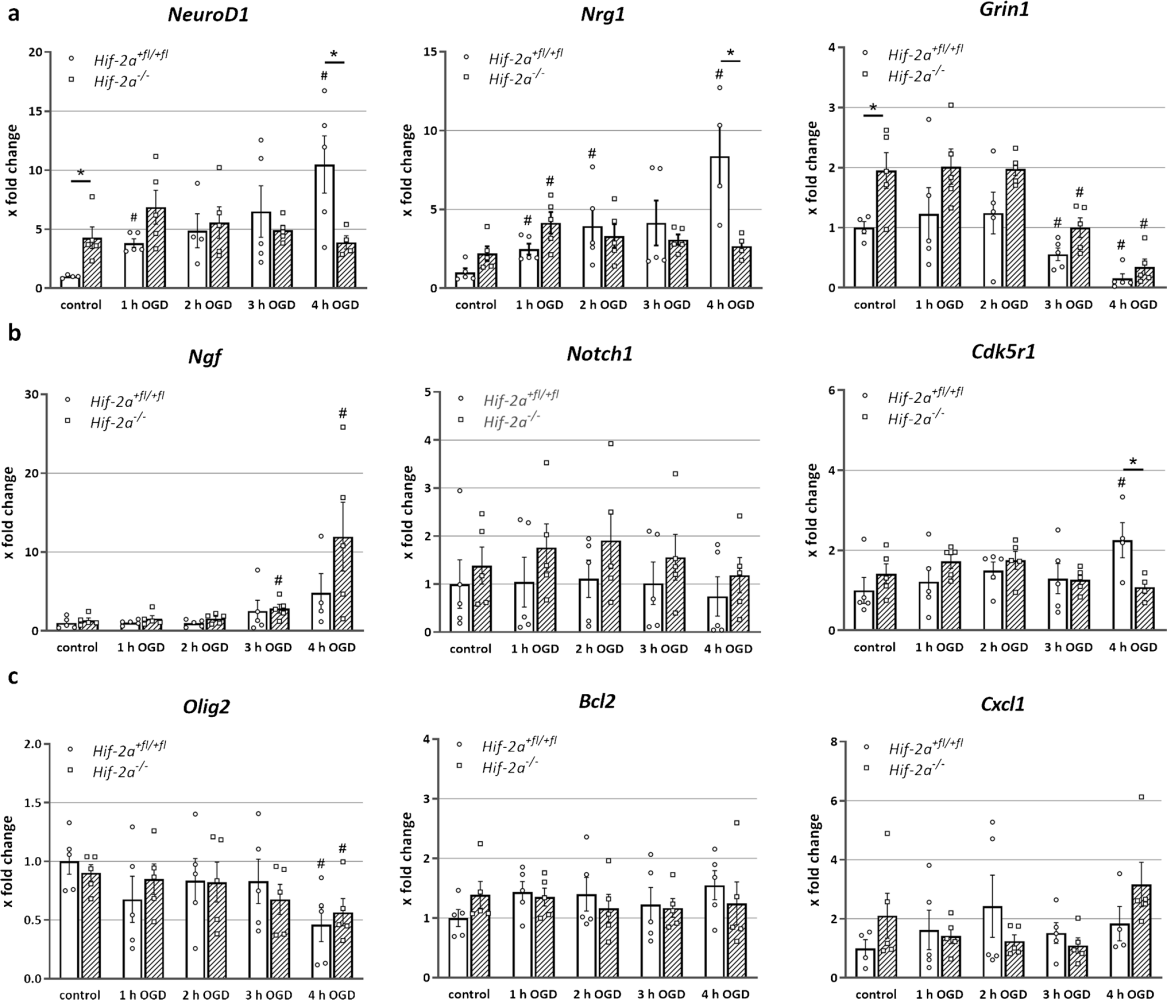
HIF-2α influences neuronal differentiation. Quantitative PCR analysis for genes related to cell differentiation and apoptosis. RNA from cells which migrated for 24 h after OGD was used. Genes were quantified and normalized for *Rpl13a* expression. **a** Neuron related genes. **b** Astrocyte- and apoptosis-related genes. **c** Oligodendrocyte-related genes. Data are presented as mean ± SEM; *p < 0.05; n = 5 with individual data points of each mouse

As the composition of the neural cells is affected differently upon OGD in KO and wild-type neurospheres, we performed expression analyses for genes known to be associated with neuronal differentiation and survival. First, we looked for *NeuroD1* expression, which is essential for neuronal differentiation. *NeuroD1* was expressed four times higher after loss of HIF-2α in the control spheres (Fig. 4a). However, OGD led only to an increase in the proficient *Hif-2α*^+*fl/*+*fl*^ spheres, whereas the KO remained at control levels. Expression of *Nrg1*, also important for neuronal differentiation, showed a constant increase in *Hif-2α*^+*fl/*+*fl*^ during OGD, compared to only a transient increase in *Hif-2α*^*−/−*^ after 1 h of OGD. *Grin1* is crucial for synaptic plasticity and survival. Its expression showed a simultaneous decrease in both genotypes after 3 h of OGD. Interestingly, initial *Grin1* expression under normal culture conditions was significantly higher in *Hif-2α*^*−/−*^ compared to the wild-type.

Another candidate for maintaining neurons is *Ngf*. Its expression levels increased steadily with ongoing OGD in both genotypes (Fig. 4 b). However, only *Hif-2α*^*−/−*^ cells showed a significant increased *Ngf* expression after 3 and 4 h of OGD. The role of *Ngf* in differentiation of neural stem cells does seem to be acute but represents a process of longer stimulation to have an additive effect on neural differentiation [7]. In our experimental design, we investigated the acute reaction of neural cells to the ischaemic event (up to 4 h OGD), which is too short a time for an effect of the NGF (nerve growth factor) protein on neuronal differentiation. However, NGF has a major influence on neuroprotection as it appears globally neuroprotective to the developing brain in a neonatal model of cerebral hypoxia–ischaemia [19]. *Notch1* is responsible for the differentiation of NSCs into astrocytes. During OGD, expression in *Hif-2α*^+*fl/*+*fl*^ cells remained at control levels and showed only a mild transient increase in *Hif-2α*^*−/−*^ spheres. *Cdk5r1* describes a gene whose expression is essential for the maintenance of neuronal cells [47]. During OGD, a significant increase in *Cdk5r1* expression was only seen in the wild-type spheres after 4 h of OGD. The expression of the *Bcl2* gene, which ensures maintenance of neurons, astrocytes and oligodendrocytes, stayed at control levels in both genotypes during OGD (Fig. 4c). *Cxcl1*, which is important for proliferation of OPCs, was unaffected during OGD. However, *Olig2* decreased significantly after 4 h of OGD in both genotypes.

Thus, we showed that HIF-2 has profound effects on gene expression during ischemic events.

## Discussion

Within this study, we showed that essential functions of brain regeneration are influenced by positive effects of HIF-2 on neural differentiation and that HIF-2 might be of more importance for regeneration after a stroke than HIF-1.

Here, we showed that migration under normoxic conditions without HIF-2α is significantly restricted (Fig. 1) confirming previous work [20]. This suggests that HIF-2α plays a crucial role in brain development to form complex brain structures. Across the neocortex, dozens of functionally different areas exist, like the primary visual area or the olfactory bulb. As neurogenesis is limited to a few areas in the brain, such as the SVZ, cells have to migrate from this region into these distinct areas [20]. Reduced viability causing the migration deficit after loss of HIF-2α was ruled out by LDH measurement that showed increased viability without functional HIF-2. This is most likely caused by elevated HIF-1α stabilization during OGD in the *Hif-2α*^*−/−*^ spheres. Investigation of familiar HIF target genes has shown that these are differentially regulated in the brain than in other organs. One example is VEGF, which plays an essential role in the formation of new blood vessels [13]. We found that *Vegf* is expressed significantly higher in *Hif-2α*^+*fl/*+*fl*^ than in *HIF-2α*^*−/−*^ (Fig. 2 b) indicating HIF-2 dependent expression of *Vegf* in the brain. However, these changes in *Vegf* expression were not found in a conditional *Hif-2α* knockout in neurons nor in a similar *Hif-1α* knockout [3]. Since hypoxic induction of *Vegf* takes place mainly in astrocytes [39], the lack of an effect of either HIF-1 or HIF-2 loss in neurons is not surprising.

HIF-2-induced *Vegf* expression in the brain might lead to new blood vessel formation, which would compensate for oxygen deficiency after a stroke. Since this is more of a long-term process, acute cell death can probably not be prevented in this way. One might assume that this represents more of a method to restore blood supply in regenerating tissue.

Overall, it should be mentioned that the general regulation of HIF target genes in the brain such as *Ldha, Cdkn1a* and *Cited2* shows a different pattern than in most other tissues that have been studied previously [9]. This includes different control mechanisms of HIF via FIH and PHDs as well as HIF target genes such as *Epo* and *Vegf.* All these findings describe the highly complex mechanism of HIFs and their influence on cellular response to hypoxic stress and regeneration of ischemic damaged tissue.

**Fig. 5:**
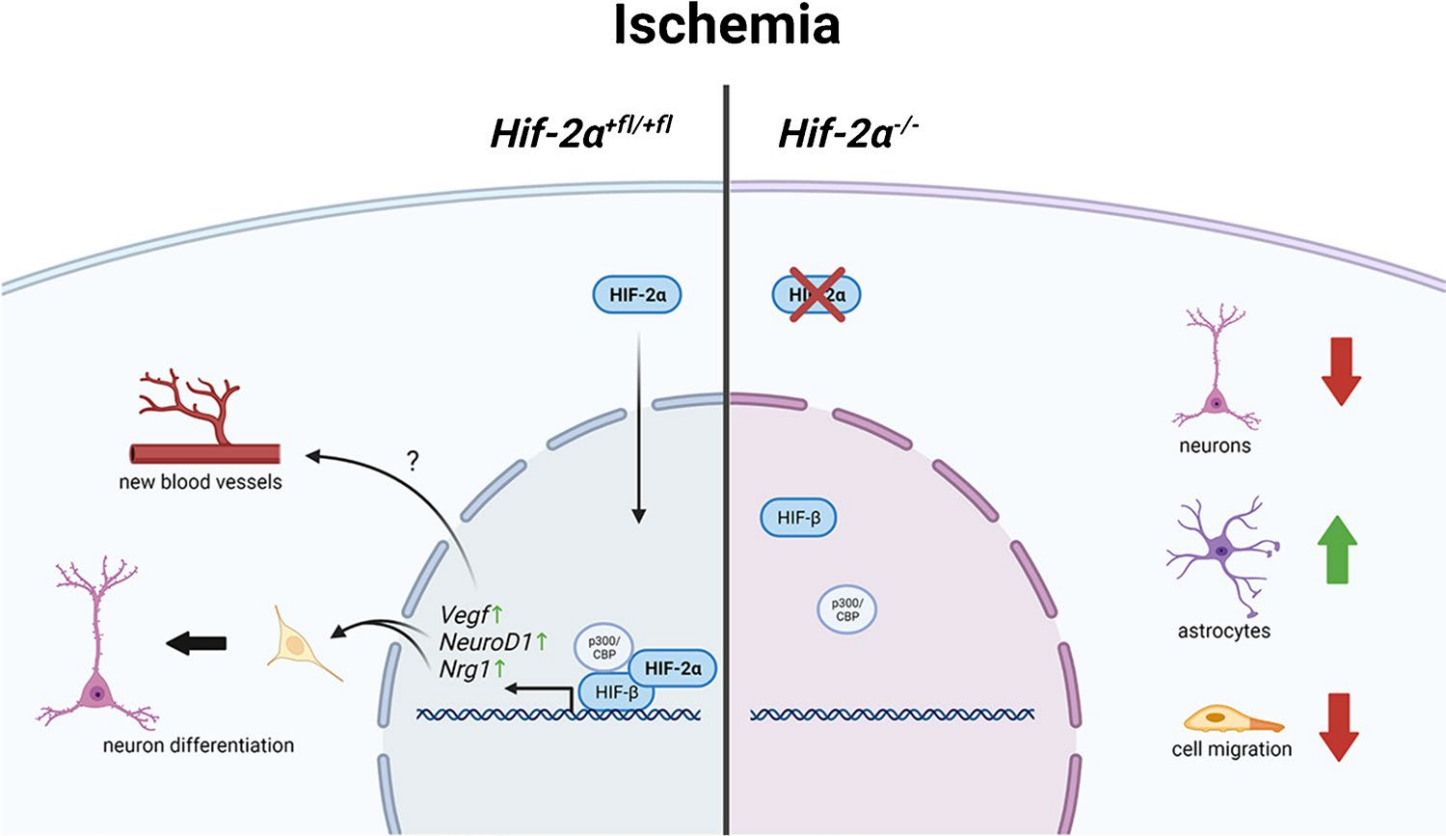
Neuroregenerative mechanism driven by the hypoxia-inducible factor 2. In animals with functional HIF-2, OGD led to an induction of *Vegf, NeuroD1* and *Ngf* expression, thereby increasing the differentiation of neuronal progenitor cells towards neurons. In combination with the increased *Vegf* expression, this might lead to improved regeneration after stroke. Upon loss of HIF-2, the number of differentiated neurons and cellular migration decreased, whereas the number of astrocytes increased. Thus, HIF-2 stabilization might prove to be valuable target for post-stroke regenerative therapy. Figure created with biorender.com

The relative number of neurons decreases significantly more in *Hif-2α*^*−/−*^ than in *Hif-2α*^+*fl/*+*fl*^ during OGD (Fig. 3b). Thus, HIF-2 may have an important function in proliferation, differentiation and/or cell survival of neurons (Fig. 5). In the case of a stroke, this would mean that HIF-2 presumably accumulates specifically in the brain in order to support differentiation of new neurons and thus promote regeneration of damaged tissue. A specific *Hif-2α* knockout in neurons, on the other hand, did not reveal any differences in infarct size compared to control after a stroke in mice [3]. One can presume that the effects of HIF-2 in neurons are not sufficient on their own to compensate for cell loss in the damaged tissue. This fact strengthens the hypothesis that an additional induction of the astrocyte number is necessary to support migrating neurons and their integration into the infarct area. Thus, for successful regeneration of the brain, not only HIF-2 in neurons, but also in astrocytes, is necessary.

A significantly increased number of astrocytes was counted after OGD (Fig. 3b). As *GFAP* is also expressed in neuronal progenitors, the observed reduction in GFAP might reflect a loss in neuronal progenitors. However, the data obtained in this study are based on manual cell counts of the immunoreactive cells. Due to the distinctly different morphologies between neural progenitor cells and astrocytes, a false count was largely avoided. One trigger for this phenomenon could be the higher gene expression of *Notch1* since the Notch signalling pathway is responsible for the differentiation of NSCs into astrocytes (Fig. 4b). As a result, proteins, generated by astrocytes, are produced at higher levels, and the effects of astrocyte-associated processes are enhanced. These include the acute erythropoietic response to hypoxic stress, which serves as neuronal protection [44]. However, the analyses of *Epo* in this study showed that although its expression was significantly stimulated during OGD, no difference between *Hif-2α*^+*fl/*+*fl*^ and *Hif-2α*^*−/−*^ was seen (Fig. 2b). It can be assumed that *Epo* in the brain is not primarily regulated by HIF-2 but by other factors, such as HIF-1. From other cell culture studies and in vivo mice analyses, *Epo* expression in hepatocytes represents a HIF-2-regulated gene [37, 43]. Preconditioning experiments showed that EPO is a main factor in mediating hypoxic adaptation through its protective effect on subsequent cerebral ischaemia [32]. Also, clinical studies suspected a protective role of EPO [10], but a later multicentre phase II/III trial did not support this hypothesis [11].

To further clarify the variations in cell differentiation, expression analyses of genes that are known to have an important influence on these cell properties were carried out. We observed a significantly higher *NeuroD1* expression in *Hif-2α*^+*fl/*+*fl*^ after 4 h of OGD compared to *Hif-2α*^*−/−*^ cells; this suggests that HIF-2α is essential for differentiation and thus new formation of neurons after ischaemic damage (Fig. 4a). This is supported by the fact that protein neurogenic differentiation 1 (NEUROD1) possesses a decisive function for neuronal differentiation and maturation (Fig. 5) [15]. In vivo enforcement of transcriptional factors, such as NEUROD1, successfully induced ectopic neuronal cells in the ipsilateral cerebral cortex and lateral striatum of the post-stroke mice brain [46] and worked as inducers to convert somatic cells into neuronal cells [17, 34, 41]. A subsequent analysis of *Nrg1* showed an expression pattern similar to *NeuroD1.* The dependence of proliferation on neuregulin-1 (NRG1) has already been shown in murine embryonic NSCs [24]. Furthermore, NRG1 signalling was highlighted as neuroprotective upon ischaemic lesion both in vitro and in vivo [29, 40]. Maintenance and survival of the neurons, on the other hand, are probably not regulated by HIF-2α. Expression of glutamate (NMDA) receptor subunit zeta-1 (*Grin1*) as a result of OGD and migration is significantly reduced in the control, but there was no difference between *Hif-2α*^+*fl/*+*fl*^ and *Hif-2α*^*−/−*^ after OGD treatment (Fig. 4 a). GRIN1 is important for connectivity between cells and the maintenance of neurons [1, 23]. Nevertheless, it has been shown that the overexpression of NMDA receptor is more likely to initiate cell death of neurons than to maintain it [1]. Therefore, the reduced expression of *Grin1* as a result of OGD could represent a protective mechanism that promotes neuronal survival at the expense of cell communication.

Translated to the treatment of stroke patients, these insights could help to improve the post-stroke outcome. A boosted stabilization of HIF-2α could support the regeneration of damaged brain tissue by promoting proliferation and differentiation of neurons. These newly generated neurons would potentially be able to compensate for the cell loss suffered. However, neurons that have migrated into the affected brain area are only capable of survival to a limited extent, as they presumably lack the adequate support of neighbouring astrocytes. In this context, it is interesting that people living at high altitude, such as in the Tibet Autonomous Region, who are permanently exposed to hypoxia, rank among the top incidence of stroke in China. Changes in the *HIF-2α* gene have been well-described in Tibetans and associated with the milder increase in haemoglobin compared to lowlanders [4]. Still, there are both gain-of-function and loss-of-function changes reported in *HIF-2α*, and genetic adaptation to high altitude relies on more than one gene. Elevated haemoglobin levels and vascular abnormalities in Tibet may contribute to the increase in blood viscosity and facilitate the formation of tiny thrombi in the cerebral circulation system [5, 6]. For both reasons, highlanders have a higher risk of stroke and a worse outcome, because hypoxia next to higher blood viscosity might damage endothelial cells and activate inflammatory responses [5, 6]. Potentially, the reduced HIF-2 activity with respect to erythropoiesis also reduces the protective function of HIF-2 in the brain.

By studying the effect of HIF-2 on astrocytes in ischaemia more closely, the performed analyses highlighted HIF-2 as a regulator for the differentiation of adult astrocytes (Fig. 3). A targeted destabilization of HIF-2, by, e. g. PHDs, would probably increase the astrocyte count and provide migrating neurons with the supporting basis to integrate into the existing cell network. This could increase the survival rate of immigrated cells and promote the regeneration of damaged tissue. However, it is quite possible that the reduction of HIF-2 levels could cause other problems, such as increased formation of glial scars, because the tasks and functions of HIF and its target genes are highly complex. The other way around, PHD inhibition seems to be an effective method to promote neuroprotection [27, 30, 38]. In particular, PHD2, as the most abundant HIF regulator in the brain, is a promising target for stroke treatment [33]. Recently, a PHD inhibitor called roxadustat (FG-4592) was approved to treat anaemia by increasing EPO production. In brain research, therapeutic approaches also focus on oral PHD inhibitors [18, 47]. The PHD inhibitor FG-4497 displayed a promising potential in preventing neuronal damage and vascular leakage after a stroke [35]. Additionally, GSK360A decreased post-stroke brain injury as well as sensory, motor and cognitive behavioural deficits in rats [47]. Nevertheless, an uncontrolled or widespread inhibition of HIF may promote regeneration in some areas while potentially damaging cells in others. In squamous epithelial cells in head and neck tumours, it has already been shown that EGFR activation via HIF-2α leads to more aggressive tumour growth, which in turn can be associated with increased motility and migration ability [25]. However, the HIF signalling pathway, and in particular HIF-2, can be seen as a valuable candidate for developing new therapeutic options for stroke patients.

## Conclusion

It should be noted that HIF-2 in the brain performs functions which in part are fundamentally different from those previously known from other tissues. There is an interplay between HIF-1 and HIF 2 in the regulation of known target genes. Essential functions of brain regeneration seem to be influenced by the positive effect of HIF-2 on neuron differentiation by means of targeted gene regulation under hypoxic conditions. These processes are likely based on complex signalling pathways such as the Notch signalling pathway. However, the repair mechanisms triggered by HIF-2 are perhaps limited and focus more on fine-tuning the neural network. Based on the findings in this study, we hypothesize that HIF-2 stabilization after a stroke is beneficial during migration and differentiation, whereas HIF-2 stabilization during establishment and survival could be damaging. Therefore, the critical time window for administering the drug needs to be established carefully. Potential therapeutic approaches include the controlled inhibition of PHDs to increase HIF-2 accumulation, thus promoting the neuroprotective effects of the HIF-2 signalling pathway.

## Data Availability

Not applicable.
